# A Trial of Fifty Years

**Published:** 1872-11

**Authors:** 


A Trial of Fifty Years.—The New York *Observer* has passed through the ordeal, and starts out anew on the second fifty years with a larger list of readers and more numerous friends than ever. Such a steady course of prosperity is unexampled, and inspires confidence. We heartily rejoice in the great success of a paper which has always advocated those sound principles that underlie the foundations of society and good government. Orthodox in the truest sense, both in Church and State, its influence is always good. We see its publishers propose to give to every subscriber for 1873 an appropriately embellished *Jubilee Year-Book.* Those who subscribe will have no cause to regret the step. $3 a year Sidney E. Morse & Co., 37 Park Row, New York.

